# Neuronal Ca^2+^-Activated K^+^ Channels Limit Brain Infarction and Promote Survival

**DOI:** 10.1371/journal.pone.0015601

**Published:** 2010-12-30

**Authors:** Yiliu Liao, Ase-Marit Kristiansen, Cecilie P. Oksvold, Frode A. Tuvnes, Ning Gu, Elise Rundén-Pran, Peter Ruth, Matthias Sausbier, Johan F. Storm

**Affiliations:** 1 Pharmacology and Toxicology, Institute of Pharmacy, University of Tübingen, Tübingen, Germany; 2 Centre of Molecular Biology and Neuroscience, and Institute of Basal Medical Sciences, University of Oslo, Oslo, Norway; National Institute on Aging Intramural Research Program, United States of America

## Abstract

Neuronal calcium-activated potassium channels of the BK type are activated by membrane depolarization and intracellular Ca^2+^ ions. It has been suggested that these channels may play a key neuroprotective role during and after brain ischemia, but this hypothesis has so far not been tested by selective BK-channel manipulations *in vivo*. To elucidate the *in vivo* contribution of neuronal BK channels in acute focal cerebral ischemia, we performed middle cerebral artery occlusion (MCAO) in mice lacking BK channels (homozygous mice lacking the BK channel alpha subunit, BK^−/−^). MCAO was performed in BK^−/−^ and WT mice for 90 minutes followed by a 7-hour-reperfusion period. Coronal 1 mm thick sections were stained with 2,3,5-triphenyltetrazolium chloride to reveal the infarction area. We found that transient focal cerebral ischemia by MCAO produced larger infarct volume, more severe neurological deficits, and higher post-ischemic mortality in BK^−/−^ mice compared to WT littermates. However, the regional cerebral blood flow was not significantly different between genotypes as measured by Laser Doppler (LD) flowmetry pre-ischemically, intra-ischemically, and post-ischemically, suggesting that the different impact of MCAO in BK^−/−^ vs. WT was not due to vascular BK channels. Furthermore, when NMDA was injected intracerebrally in non-ischemic mice, NMDA-induced neurotoxicity was found to be larger in BK^−/−^ mice compared to WT. Whole-cell patch clamp recordings from CA1 pyramidal cells in organotypic hippocampal slice cultures revealed that BK channels contribute to rapid action potential repolarization, as previously found in acute slices. When these cultures were exposed to ischemia-like conditions this induced significantly more neuronal death in BK^−/−^ than in WT cultures. These results indicate that neuronal BK channels are important for protection against ischemic brain damage.

## Introduction

Following focal cerebral ischemia, neuronal death is caused by both necrosis in the core and apoptosis in the penumbra within minutes to days [1]–[3]. Although multiple factors are involved, disturbance of the neuronal ionic homeostasis is likely to be fundamental for these processes. Dissipation of ionic gradients during ischemia causes depolarization that opens voltage-gated Ca^2+^ channels and unblocks NMDA-type glutamate receptor channels. Ischemic energy depletion leads to Ca^2+^ pump and Na^+^/K^+^ pump failure that elevates intracellular [Na^+^] and can reverse the Na^+^/Ca^2+^ exchanger, further increasing [Ca^2+^]_i_. Elevation of Ca^2+^ in synaptic terminals releases excitatory amino acids (EAA) [4], [5], which activate AMPA-, NMDA- and metabotropic glutamate receptors, thus triggering Ca^2+^ influx and release from internal stores. Such Ca^2+^ accumulation and excitation during ischemia drastically reduced neuronal energy production causes cell death through excitotoxicity [6], [7]. The severely elevated [Ca^2+^]_i_ leads to excessive Ca^2+^-accumulation in mitochondria, causing release of free radicals and apoptotic factors^4^, and activates Ca^2+^-dependent enzymes which degrade macromolecules [8]–[10].

In this critical situation, there is a need for a mechanism that can be activated by the elevated [Ca^2+^]_i_ and depolarization, and suppresses harmful, energy-consuming discharge activity, Ca^2+^ influx, and transmitter release. Opening of the BK type, large-conductance voltage- and Ca^2+^-gated potassium channel repolarizes and hyperpolarizes the neuron by driving the membrane potential towards the K^+^ equilibrium potential. Therefore, it may cause negative feed-back-regulation of Ca^2+^ influx and neurotransmitter release, and may thus constitute a powerful ‘brake’ on cell excitation, Ca^2+^ accumulation, and excitotoxicity [11]–[17].

A previous attempt to test this hypothesis *in vivo* [18] was inconclusive because the BK channel opener used (BMS-204352), proved to be a potent opener of Kv7 potassium channels [19]–[21]. In hippocampal slice cultures, it was found that BK channel blockers [22] aggravated cell damage following oxygen and glucose deprivation. However, *in vitro* mechanisms may differ from those operating *in vivo*. Furthermore, some BK channels are rather resistant to iberiotoxin [23] and paxilline may not be entirely BK channel-selective [14]. Finally, although BK channels were previously believed to be unequivocally inhibitory, reducing neuronal excitability [24], [25], we and others have recently found that they can actually facilitate neuronal firing [26], [27] and promote epilepsy [28], [29]. These complicating factors make it hard to predict the impact of BK channels on ischemic pathogenesis. Here, by using homozygous BK channel-deficient mice, in which the gene encoding the pore-forming BK channel subunit was inactivated (BK^−/−^ mice) [27] we find that neuronal BK channels are neuroprotective during acute ischemic stroke, profoundly limiting brain damage and promoting survival. Preliminary reports of these results were previously presented in abstract form in 2006 and 2009 [30], [31].

## Materials and Methods

### Middle cerebral artery occlusion (MCAO) model of ischemic stroke

BK channel-deficient (BK^−/−^) and wild-type (WT) mice [27], either litter- or age-matched (11–14 weeks) males with hybrid SV129/C57BL6 background (always F2 generation) were anesthetized by isoflurane and placed on a heating pad during surgery. The right common carotid artery (CCA) was isolated. All branches of the right external carotid artery (ECA) were electro-coagulated. After isolation of the CCA-internal carotid artery (ICA) axis and the CCA-ECA axis, both sutures surrounding the proximal common and distal internal carotid arteries were lightly drawn to disrupt blood flow transiently. A 5-0 monofilament nylon suture –heat-rounded at its tip- was inserted through a small incision into the ECA via the external carotid stump and into the proximal ICA, and through the cranial base. A slight resistance indicated that the tip was lodged in the anterior cerebral artery and blocked blood flow. The occluding suture was inserted in the intracalvarium. After the correct placement, the ECA stump was cauterized to avoid bleeding. After onset of the middle cerebral artery occlusion, all sutures for control of the proximal and distal CCA were immediately removed and the skin was closed. After an occlusion of 90 minutes, the skin was re-opened, the occluding suture was removed and the ECA stump was closed with a 7-0 silk suture to avoid bleeding. Wounds were closed with a 5-0 suture, and mice were kept under a heating lamp until they recovered.

Regional cerebral blood flow (rCBF) measurements were performed during anesthesia (pre-ischemic status), 10 minutes after onset of MCAO and 10 minutes after reperfusion, via a 2.5-mm-hole drilled 1.5 mm lateral to the midline and 2 mm posterior to the bregma. Preliminary experiments showed that this location was recruited into infarction. The Laser Doppler (LD) probe connected to a LD flowmeter was positioned stereotactically. LD measurement monitors the red blood cell flux through microvasculature (number of red blood cells in the tissue sampling volume times their mean velocity), recorded in blood perfusion units (BPU) on a relative scale [32]. The rCBF data were recorded and analyzed using a MacLab 4/20 system.

### Intracerebral NMDA application, analysis of brain infarctions

The skull of anesthetized mice was exposed, and a hole (2.5 mm in diameter) was bored 1.5 mm caudal to bregma and 4.0 mm from the midline [33]. 500 nl of a solution containing 50nmol NMDA was stereotactically injected into the parietal cortex 1.5 mm below the dural surface using a micro-liter syringe. 24 hours later, neurological deficits were evaluated. The neurological deficit after MCAO or NMDA application was assessed blindly by two investigators using a four-point scoring system established for rats [34], [35] and mice [33], [36].

#### TTC staining

Brains were immersed in 4°C isotonic NaCl solution and posted in cooled agar gel. Consecutive coronal 1-mm-sections were performed using a McTwain tissue chopper (Mickle Laboratory Engineering Co. Ltd, Surrey, UK) and incubated for 20 min at RT in 2% 2,3,5-triphenyltetrazolium chloride (TTC). Brain slices were post-fixed at 4°C in 2% paraformaldehyde overnight, and photographed in a microscope.

#### Quantification of infarctions

Quantification of infarctions and NMDA lesion areas were performed using Image Tool for windows 2.0 (University of Texas Health Sciences Center) and expressed as percentage of the ipsilateral hemisphere. Lesion and infarction volumes were calculated by integrating the infarction areas of all sections and their thickness of 1 mm [37].

### Organotypical hippcampal slice cultures

were prepared as described [38]. WT and BK^−/−^ pups were sacrificed at postnatal days 6 or 7 (p6-p7). Brains were placed in a medium containing (in mM): 137 NaCl, 5.0 KCl, 0.85 NaH_2_PO_4_, 1.5 CaCl_2_, 0.22 KH_2_PO_4_, 0.28 MgSO_4_, 1.0 MgCl_2_, 2.74 NaHCO_3_ and 45 glucose, dissolved in tissue grade water. Both hippocampi were dissected out and cut into 400 µm thick transverse slices using a McIlwain tissue chopper. Only the central part of each hippocampus was used. Slices were put on filter membranes (0.4 µm Hydrophilic PTFE filters, Ø30 mm, Millipore), and cultivated in 6-wells plates at 36°C for 20–21 d. Each well was supplied with 1 ml medium consisting of Basal Medium Eagle with HBSS (50%) and Hanks balanced salt solution, HBSS (25%) (both from AMIMED), heat inactivated horse serum (25%), penicillin/streptomycin (100 U/ml) and 1 L-glutamine, 20 glucose and 6 NaHCO_3_. The medium was first changed after 24 h, and then every 3–4 days.

#### Whole-cell patch clamp recordings

Hippocampal slice cultures (14–20 days *in vitro* (DIV)) were perfused (submerged) with artificial cerebral spinal fluid (ACSF) containing (mM): 125 NaCl, 25 NaHCO_3_, 2.25 KCl, 1.25 KH_2_PO_4_, 2 MgCl_2_, 2 CaCl_2_, 16 glucose, and saturated with a gas mixture containing 95%O_2_/5%CO_2_. Whole-cell somatic patch clamp recordings from CA1 pyramidal cells were performed with an Axoclamp 2A amplifier and pipettes filled with 140 KMeSO_4_ or K-gluconate, 10 HEPES, 10 phosphocreatine-Na, 2 ATP-Na, 0.4 GTP-Na, and 2 MgCl_2;_ pipette resistance: 4–7 MΩ; series-resistance: 10–40 MΩ. Potentials were corrected for the junction potential. Only cells with stable resting potential <−60 mV and spike ampliudes >70 mV were used.

#### In vitro ischemia (IVI)

IVI was induced after 20–21 DIV by exposing the culture to sterile, artificial ‘ischemic’ cerebral spinal fluid (iCSF): 70 NaCl, 70 KCl, 2 MgSO_4_, 1.25 NaH_2_PO_4_, 20 sucrose, 6 NaHCO_3_, 0.3 CaCl_2_, saturated with 95%N_2_/5%CO_2_ (pH 6.8). These conditions resemble *in vivo* ischemia [39], [40]. Cultures were pre-incubated the day before IVI with propidium iodide (PI, 5 µg/ml) to indicate cell death [22], [41]. Cultures with indistinguishable CA1, CA3 and DG regions, or with PI staining before the start of the experiment, were excluded. IVI was induced by moving the cultures (via two iCSF-PI washing steps) to iCSF with PI and exposing the cultures to IVI for 13–17 min (36°C), and then (via two washing steps) back into normal medium with PI at 5% CO_2_. For control, slice cultures were incubated for 13–17 min with sterile ACSF: 125 NaCl, 2.5 KCl, 2 MgSO_4_, 1.25 NaH_2_PO_4_, 20 glucose, 25 NaHCO_3_, 2 CaCl_2_ and PI, saturated with 95%O_2_/5%CO_2_, pH 7.4).

#### Quantification of IVI-induced cell death

Quantification of IVI-induced cell death was based on the observation that PI fluorescence correlates linearly with histologically defined neuronal damage and cell death [22], [41].

### Approval

The *in vitro* experimental procedures were approved by the responsible veterinarian (ethical committee) of the Institute of Basic Medical Sciences at the Faculty of Medicine, University of Oslo, in accordance with the statute regulating animal experimentation, given by the Norwegian Ministry of Agriculture, 1996. The approval for the *in vivo* stroke experiments was obtained at 5.12.2006 from the ethics committee of Tübingen (Regierungspräsidium AZ 35/9185.81-2), Research-Nr. PZ1/06.

### Statistics

Infarct volumes, NMDA lesion volume, neurologic deficit scores, reduction of blood flow were compared using two-tailed Student's t-test. Infarct and NMDA lesion area comparisons were made by Kruskal-Wallis test followed by Mann-Whitney *U* test. Statistical analysis of the IVI data was performed with SPSS (version 13.0). A General Linear Model was used to compare the time course of cell death in the experimental groups in the IVI experiment, followed by Bonferroni's post hoc correction.

## Results

### High mortality, severe neurological deficits, and increased infarction volume in BK^−/−^ mice after middle cerebral artery occlusion

**Figure 1:**
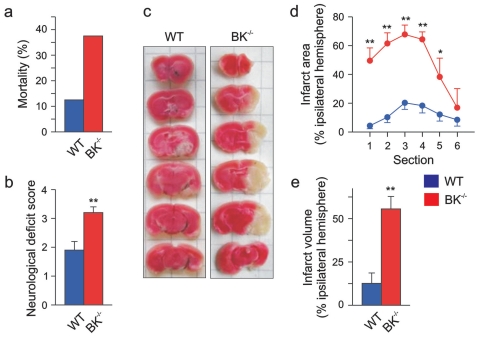
Targeted deletion of BK channel increases mortality and severity of brain infarction after transient middle cerebral artery occlusion. (**a**) Mortality within 7 h after reperfusion (in 8 WT vs. 8 BK^−/−^ mice tested), and the neurological deficit score (**b**) evaluated 7 h after reperfusion from seven WT and five BK^−/−^ mice. (**c**) Representative photomicrographs from coronal WT and BK^−/−^ brain sections (1 mm thick, from rostral (top) towards caudal (bottom)) stained with TTC 7 h after reperfusion. Lack of red staining indicates infarction. The infarction area of every brain section (**d**) and the resulting infarction volume of the ipsilateral hemisphere (**e**) are plotted. All data are given as mean ± SEM; WT: *n* = 7, BK^−/−^: *n* = 5; *P<0.05, **P<0.01.

To evaluate the role of BK channels in acute focal cerebral ischemia, we performed middle cerebral artery occlusion [42] in WT and BK^−/−^ mice. Occlusion for 90 minutes followed by a 7 hours reperfusion period caused a 3-fold higher mortality in BK^−/−^ mice (3 of 8 mice died) compared to WT (1 of 8) (**Fig. 1a**). In addition, the surviving five BK^−/−^ mice showed more severe neurological deficits than the WT mice (neurological deficit score BK^−/−^ 3.2±0.2 vs. WT 1.9±0.3) (**Fig. 1b**). In a preliminary study, with 120 min occlusion and 24 h reperfusion, 5 of 6 BK^−/−^ mice died during the reperfusion period while only 1 of 6 WT mice died during the reperfusion period (data not shown).

**Figure 2:**
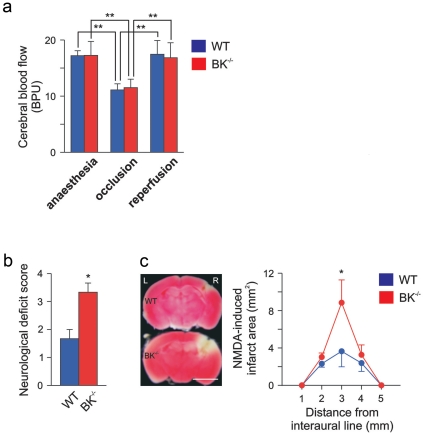
Cerebral blood flow and NMDA-induced excitotoxicity in WT and BK^−/−^ mice. (**a**) Cerebral blood flow (given as blood perfusion units [BPU], see also *Methods*) was measured with laser Doppler flowmetry during anaesthesia (pre-ischemia), 10 min after onset of occlusion (intra-ischemia) and 10 min after reperfusion onset (post-ischemia), and is presented as mean ± SEM from 7 WT and 5 BK^−/−^ mice; **P<0.01 (One-way ANOVA, followed by Tukey's post hoc analysis). (**b**) The neurological deficit score was evaluated 24 h after an intra-cerebral microinjection of 50 nmol of a NMDA solution in a volume of 500 nl. (**c**) *Left:* Representative WT and BK^−/−^ brain sections stained with TTC show the lesion area 24 h after NMDA-microinjection; bar: 4 mm. *Right:* The corresponding statistic based on the analysis of 3 WT and 3 BK^−/−^ mice. Data are given as mean ± SEM; *P<0.05.

The severe neurological deficit was accompanied by a far larger ischemic brain infarct in BK^−/−^ compared to WT mice, as measured by 2,3,5-triphenyltetrazolium chloride (TTC) staining. (infarcted area: BK^−/−^ 55.6±7.9%, WT 12.6±6.0%, n = 5–7; p<0.001) (**Fig. 1c-e**). The infarct was wide-ranged, reaching from basal ganglia to cerebral cortex in BK^−/−^, whereas mainly basal ganglia were affected in WT mice (**Fig. 1c**). To avoid methodological artifacts, the same occluding suture with its heat-rounded tip was used throughout all MCAO experiments. Since we found no anatomical abnormalities in the brain of BK^−/−^ mice [27], [43], these observations indicate that BK channels are highly important for survival and functional recovery after acute ischemic stroke. However, since these channels were constitutively deleted in BK^−/−^ mice [27], this type of experiments cannot distinguish between the possibilities that vascular or neuronal BK channels, or both, mediate the protection. Thus, the increased infarction volume in BK^−/−^ mice after MCAO might conceivably be due to increased contractility of the middle cerebral artery or its branches, causing a reduced reperfusion, since BK channel deletion causes an increased vascular tone of small arteries [44]. Measurement of regional cerebral blood flow before, during, and after onset of reperfusion by LD flowmetry (**Fig. 2a**), however, revealed no differences in cerebral blood flow between the genotypes. Thus it appears that the increased infarct volume in BK^−/−^ mice was not caused by haemodynamic differences during the ischemic insult, although BK channels are important also for opposing vasoconstriction.

### Neuronal BK channel protects against NMDA excitotoxicity

To test whether lack of neuronal BK channels aggravates ischemic damage, we induced glutamate receptor-dependent excitotoxicity by intracerebral application of NMDA, mimicking an excessive pre-synaptic glutamate release. Twenty-four hours after NMDA microinjection, the BK^−/−^ mice showed a worse neurological deficit score than the WT mice (BK^−/−^ 3.2±0.2; WT 1.8±0.2) (**Fig. 2b**) correlating with the increased infarction area found in brain sections (**Fig. 2c**) and larger infarction volume (WT 4.8±2.1 mm^3^, BK^−/−^ 10.8±1.2 mm^3^). Although the neurological deficit scores were similar in NMDA-injected compared to artery occlusion-treated BK^−/−^ mice, the lesions determined by TTC staining were strikingly different in these two sets of experiments (**Fig. 1c** **and** **2c**). Whereas NMDA-induced lesions were primiarily cortical, middle cerebral artery occlusion caused both cortical and severe deep infarctions in BK^−/−^ mutants. The different topography of two types of lesions may strongly affect the neurological outcome. Thus, the neurological outcome depends on both size and topology of the infarction area. Nevertheless, the results support the idea that neuronal BK channels protect against acute ischemic stroke by preventing NMDA-induced excitotoxicity, thus pointing to neuronal rather than vascular BK channels in neuroprotection.

### BK channels contribute to neuronal action potential repolarization and fast afterhyperpolarzation (fAHP) in an *in vitro* model for ischemic stroke

**Figure 3:**
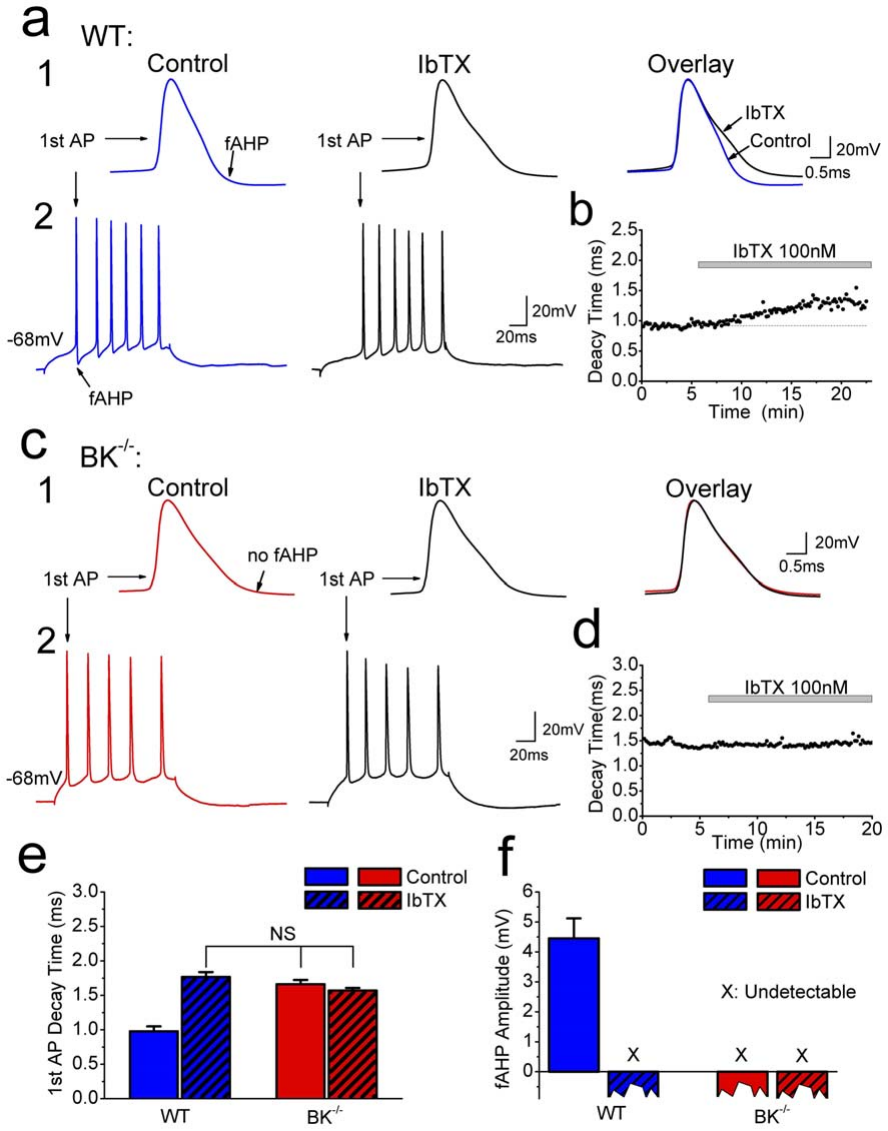
BK channels contribute to spike repolarization and fast afterhyperpolarzation (fAHP) in cultured CA1 pyramidal cells. (**a**) Whole-cell recording from CA1 pyramidal cell in WT organotypic slice culture. A 100 ms depolarizing current pulse was injected, evoking a train of action potentials (**a2**). Bath application of IbTX (100 nM) slowed repolarization of first action potential in the spike train and abolished the fAHP (**a1**). (**b**) Time course of IbTX effect on 1st action potential decay time (90%-10%) in a WT cell (same as in (a)). (**c**) Same test as in (a) applied to a BK^−/−^ slice culture (**c2**). 1st action potential was broader than in WT cells and lacked fAHP. IbTX had no detectable effect (**c1**). (**d**) Time course of IbTX effect on 1st action potential decay time (90%-10%) in a BK^−/−^ cell (same as in (c)). (**e**) Summary of 1st action potential decay time ± IbTX, in CA1 pyramidal cells from WT and BK^−/−^. Decay time of WT cells under control condition was significantly shorter than for the other three groups (n = 4 in each group, *p*<0.01). (**f**) Summary of fAHP amplitude (measured at the time point corresponding to the peak of the fAHP in WT cells). The fAHP amplitude was close to zero (not measurable) in BK^−/−^ and after application of IbTX in WT and BK^−/−^ cells.

Next, we examined the effects of neuronal BK channel ablation in a cerebral ischemia model where systemic, vascular and most other non-neuronal effects were irrelevant. Thus, in hippocampal slice cultures [38], we used a protocol for *in vitro* ischemia (IVI), which mimicks ischemic stroke [22], [39], [45], [46] and permits monitoring of long-term neuronal survival. Although it is well established in acute brain slices that BK channels contribute substantially to neuronal spike repolarization and fast afterhyperpolarzation in hippocampal CA1 and CA3 pyramidal cells [12], [17], [26], these functions have previously not been demonstrated in slice cultures. Therefore, we performed whole-cell recordings from CA1 pyramidal neurons in slice cultures (**Fig. 3**), while applying the selective BK channel blocker, iberiotoxin (IbTX, 100 nM). IbTX caused a characteristic slowing of the late phase of the spike repolarization and blocked the fAHP [16] in WT cultures (**Fig. 3a,b**), whereas it had no effect in BK^−/−^ cultures (**Fig. 3c,d**). Furthermore, the spikes of BK^−/−^ neurons were significantly broader than those of WT neurons before IbTX application (p = 0.0012, n = 4, **Fig. 3d,e**) and the fAHP was suppressed (**Fig. 3f**), demonstrating that BK channels underlie spike repolarization and fAHP of pyramidal neurons in these cultures. Since we have previously found that pharmacological suppression of BK channel-dependent spike repolarization can cause increased glutamate release in hippocampal tissue^6^, glutamate release may be enhanced in BK^−/−^ cultures.

### Aggravated neuronal death in BK^−/−^ slice cultures after *in vitro* ischemia

**Figure 4:**
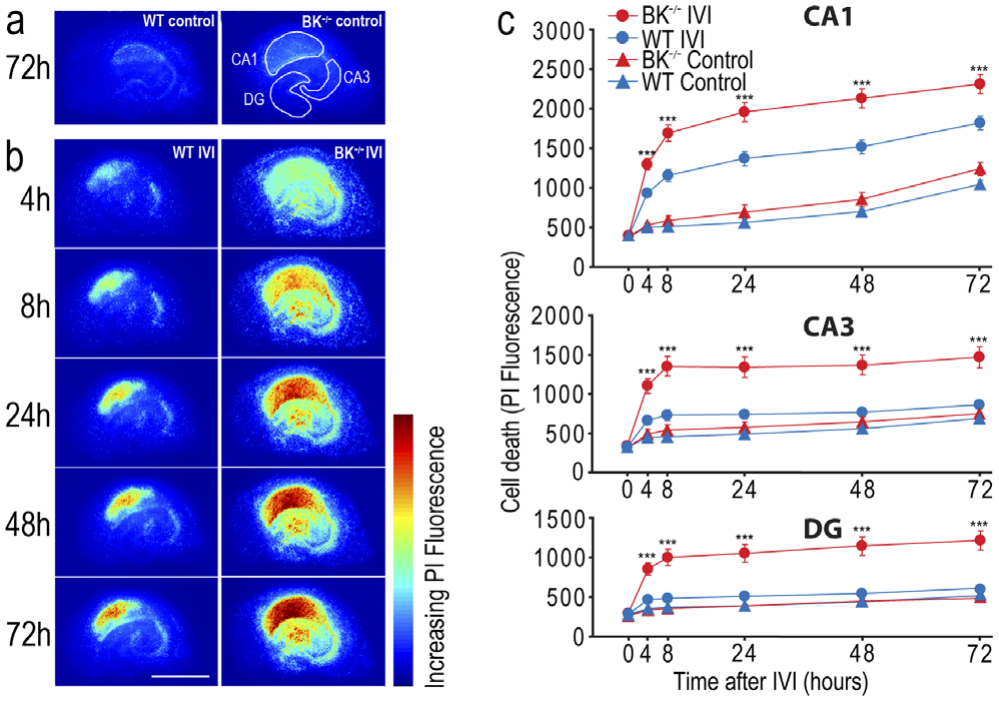
Lack of BK channels aggravates cell death in CA1, CA3 and DG after *in vitro* ischemia (IVI). (**a,b**) Representative pseudocolour images showing PI fluorescence intensity as indicator of cell death measured 4 h, 8 h, 24 h, 48 h and 72 h after IVI. (**a**) BK^−/−^ and WT cultures without IVI (control cultures) maintained for 72 h. Regions of interests (CA1, CA3, dentate gyrus (DG)) are outlined. (**b**) BK^−/−^ and WT cultures at different time points after exposure to IVI. Scale bar  = 1 mm. (**c**) Time course of cell death measured as PI fluorescence in CA1 (*upper*), CA3 (*middle*) and DG (*lower*). Data are given as mean ± SEM; WT IVI: *n* = 44, BK^−/−^ IVI: *n* = 50, WT control: *n* = 25, BK^−/−^ control: *n* = 23; ***P<0.005. Slice cultures were from a minimum of two pups per experimental set.

We subjected WT and BK^−/−^ organotypic hippocampal slice cultures to *in vitro* ischemia [39] and measured the degree of ischemic damage in subfields CA1, CA3 and dentate gyrus (DG) by using propidium iodide (PI) as indicator of cell death (**Fig. 4**). Previous results indicate that cell death in this ischemia model increases from 4 to 8 hours after IVI, with a further increase towards 48 hours [39], in accordance with the delayed cell death seen in *in vivo* models of ischemic stroke [22]. Therefore, we analyzed cell death at six different time points after IVI: 4, 8, 24, 48 and 72 hours. In WT slice cultures, we observed an increased neuronal death 4–8 h after IVI in the CA1 area. Delayed cell death was also more pronounced in the CA1 area (24, 48 and 72 h) compared to CA3 and DG. Although less than in CA1, some cell death was also found in the CA3 area and the DG of WT cultures (**Fig. 4b,c**). These results are consistent with previous IVI studies in hippocampal slice cultures from rats [22], [39].

In BK^−/−^ slice cultures, the ischemia-induced neuronal death showed the same general pattern as in WT; thus CA1 showed greater sensitivity towards ischemia than CA3 and DG. The degree of higher IVI vulnerability of CA1 compared to CA3 and DG was similar for BK^−/−^ and WT cultures as assessed by the General Linear Model (CA1 vs. CA3: F(1,92) = 0.265 n.s.; CA1 vs. DG: F(1,92) = 0.19 n.s.). However, the damage was significantly more severe for all time points (4, 8, 24, 48, 72 h) in BK^−/−^ than in WT cultures, in both CA1 (F(3,138) = 37.1, P<0.001), CA3 (F(3,138) = 16.2, P<0.001), and DG (F(3,138) = 18.6, P<0.001) (**Fig. 4b, c**). This is in agreement with the high level of BK channel expression in mouse hippocampus^27^. In CA1 the mean cell death at all time points was slightly higher in BK^−/−^ than in WT cultures even without IVI (F(1,46) = 2.65 n.s.) (**Fig. 4a,c**). These result support the idea that BK channels protect neurons against ischemia-induced cell death, thereby prolonging their survival.

## Discussion

Two decades ago, it was found that BK channels can be activated during the action potential in mammalian brain neurons, thus accelerating spike repolarization and limiting Ca^2+^ influx [11], [12], [16], [47], [48]. Because of its unique dependence on both depolarization and intracellular Ca^2+^, it was suggested that this channel type mediates negative feedback regulation of Ca^2+^ influx and transmitter release [11]. This prediction was later verified in glutamatergic synapses of the mammalian brain [17], [27]. Thus, rat hippocampal BK channels not only regulate somatic excitability, but are also specifically targeted to the active zone of presynaptic glutamatergic terminals where they can limit glutamate release under conditions of increased Ca^2+^ influx [17]. BK channels may therefore provide a neuroprotective ‘emergency brake’ when there is excessive depolarization and [Ca^2+^]_i_ accumulation, such as during cerebral ischemia [11], [17], [18], [49]. We have now for the first time tested this hypothesis directly by selective suppression of BK channel activity, using BK^−/−^ mice [27]. All our tests yielded consistent and convergent results, indicating that neuronal BK channels play an important role in neuroprotection against acute ischemic brain damage after stroke.

Over-activation of NMDA receptors can lead to neuronal cell death, as observed in post-ischemic stroke and many other acute and chronic disorders [50], [51]. In the present study, we found that NMDA receptor-induced excitotoxicity and the corresponding brain infarction were aggravated in BK^−/−^ mice versus WT. This might be partly caused by enhanced Ca^2+^ influx due to broadening of spikes in BK^−/−^ neurons (**Fig. 3b**) or lack of negative feed-back regulation of NMDA receptor-mediated Ca^2+^ influx and depolarizaton. BK channels in glutamatergic terminals may, under certain conditions, act by shortening presynaptic action potentials, thereby reducing Ca^2+^ influx, as previously shown in hippocampal and other nerve terminals [13], [17], [52]. Thus, enhanced glutamate release in BK^−/−^ mice is suggested to contribute to enhanced Ca^2+^ influx and neurotoxicity. In support of our hypothesis, Raffaelli *et al.* (2004) [53] reported that BK channel blockade caused enhanced glutamate release under basal conditions *in vitro,* and reduced oxygenation tension in acute brain slices enhances the contribution of presynaptic BK channels to regulation of glutamate release [54], [55].

Furthermore, it seems plausible that ischemia-induced depolarization may cause inactivation of voltage-gated K^+^ channels that normally dominate presynaptic spike repolarization [56]–[58], thus allowing more BK channels to activate and dominate the repolarization [48], [59]. BK channel activation could be further promoted through an ischemia-induced failure of Ca^2+^ pumping and increase in [Ca^2+^]_i_, thus enhancing the role of BK channels in limiting vesicular glutamate release and preventing depolarization-induced reversal of glutamate transporters [60]. Thus, neuronal BK channels may be well suited for negative feedback regulation of cellular excitability and neurotransmitter release, as suggested by *in vitro* studies of glutamatergic synapses [17], [53], [61].

Although potassium channels are generally assumed to be inhibitory [25], it was recently shown that BK channels can actually facilitate high-frequency burst firing in hippocampal pyramidal cells [26], promote spontaneous spiking in cerebellar neurons [27], and cause epilepsy in humans [28], [29]. Thus, on one hand, the BK channels are expected to limit the Ca^2+^ influx and glutamate release caused by each action potential; on the other hand, they may promote a higher spike frequency in some cases. Our findings that loss of BK channels aggravates the ischemic mortality, infarction and cell death *in vivo* as well as *in vitro* suggest that the former, protective actions of BK channels dominate during ischemia.

Why did the BK channels apparently not play any significant role in regulating the cerebral blood flow in the healthy brain or within the ischemic lesion? One possibility is that the arterial occlusion may alter the sensitivity of cerebral blood vessels to BK regulation, e.g. because BK channels in cerebral blood vessels may be inhibited by O_2_-deprivation [62]. Alternatively, it is possible that the effect of BK channel regulation of cerebral blood flow is so mild that laser Doppler flowmetry failed to detect a significant effect. This latter possibility is supported by our previous study, which indicated that the systemic blood pressure in BK channel-ablated mice is only moderately increased [27]. A third possibility is that the effects of blood vessel contraction in BK^−/−^ mice were neutralized by changes in vascular tone regulation during ischemia, since hemodynamic and metabolic changes in acute ischemia of CNS can cause disruption of the blood/brain barrier and dysregulation of vascular tone [63]. However, regardless of which vascular mechanisms underlie the apparent lack of haemodynamic changes, the increased stroke susceptibility following arterial occlusion in BK^−/−^ mice, along with the aggravated cell death under IVI in hippocampal BK^−/−^ slice cultures, suggest a primarily neuronal mechanism for the protective effect of BK channels limiting brain damage during ischemia.
